# Education level as a predictor of the onset of health problems among China’s middle-aged population: Cox regression analysis

**DOI:** 10.3389/fpubh.2023.1187336

**Published:** 2023-07-14

**Authors:** Ruru Ping, Takashi Oshio

**Affiliations:** ^1^Graduate School of Economics, Hitotsubashi University, Tokyo, Japan; ^2^Institute of Economic Research, Hitotsubashi University, Tokyo, Japan

**Keywords:** educational level, Cox proportional hazard model, midlife health, non-communicable disease, relative index of inequality

## Abstract

**Background:**

Despite the importance of midlife with reference to one’s health, educational inequalities in midlife health have attracted little attention in China. Using Cox proportional hazards regression analysis, this study examined the association between educational attainment and the onset of midlife health problems and investigated the potential mediating effects of socioeconomic position (SEP) other than educational attainment, depression, and health behavior.

**Methods:**

Data were extracted from the China Health and Retirement Longitudinal Survey (CHALRS) from 2011 (baseline) to 2018 (latest data). Participants aged 45–59 years at baseline were studied (*N* = 8,050). Health outcomes included the onset of poor self-rated health (SRH), limitation in activities of daily living (ADL) and instrumental ADL (IADL), multimorbidity, hypertension, dyslipidemia, heart diseases, and stroke over the 7-year follow-up period. Cox proportional hazard models were used to examine the associations of the outcomes with educational attainment, while controlling for potential mediators (other SEP, depression, and health behaviors).

**Results:**

Lower educational level was associated with increased incidences of poor SRH and ADL/IADL limitations, but with decreased incidences of dyslipidemia and heart disease. After adjusting for baseline covariates, the RII was 2.17 (95% confidence interval [CI]: 1.74, 2.70) for poor SRH, 2.15 (95% CI: 1.42, 3.26) for ADL limitation, 3.84 (95% CI: 2.98, 4.94) for IADL limitation, 0.52 (95% CI: 0.40, 0.68) for dyslipidemia, and 0.55 (95% CI: 0.40, 0.74) for heart disease. Significant proportions (2.1 to 27.0%) of the RII were explained by the mediators. No sex or urban–rural differences were found in this study.

**Conclusion:**

Our findings suggest that educational attainment is an important predictor of the incidences of key midlife health problems, with significant mediating effects exerted by other indicators of SEP, depression, and health behavior.

## 1. Introduction

Midlife is a transitional stage in the life course of human beings, and links earlier life and older adulthood, when professional and other types of growth coincide with the onset of physical decline. This period encompasses a variety of physiological and psychosocial transitions, which are pivotal for one’s health, functioning, and wellbeing (1–4). Given the importance of this stage of life, this study aims to examine educational inequalities on midlife health among the Chinese population, which is one of the world’s fastest growing ageing populations (5). Educational attainment is often used as a socioeconomic position (SEP) indicator in health studies, because within a life course framework, it reflects long-term influences of parental SEP on young adulthood SEP, which in turn represents a strong determinant of adulthood resources such as income and occupation (6). There is ample evidence implying that people with higher educational attainment are more likely to be healthier in terms of physical health, mental health, functional ability, and cognitive performance (7–9). In the context of China, there are two critical empirical gaps in existing literature on this topic: (I) How consistent is the effect of educational inequalities on health in China, especially among the middle-aged population? (II) To what extent is educational inequalities on midlife health mediated by other socioeconomic, psychosocial, or behavioral factors?

Previous studies have shown contradictory evidence on the association between educational attainment and health in China. It was found that lower educational attainment was associated with poorer self-rated health (SRH) than higher educational level among people aged ≥14 years in southern China (10), a lower risk of physical multimorbidity among Chinese aged ≥50 years (11), and a higher risk of IADL (instrumental activities of daily living) disability among Chinese aged 20–69 years (12). However, lower educational attainment was not associated with a significant disability regarding ADL (activities of daily living) (12). A lower education level was associated with an increase in the incidence of metabolic syndrome and systolic blood pressure among Chinese women, but not among Chinese men (13, 14). However, the association between educational level and the onset of diastolic blood pressure was insignificant for both sexes (14). The results from these previous studies have been inconsistent, and none of the studies focused on midlife, which is a pivotal period in a person’s life; studies focused on this period can be applied for understanding and lowering morbidity and mortality rates in the middle age and in older adulthood. To contribute to the existing literature on this topic, this study aims to assess educational attainment as a predictor of midlife health, using data from a nationwide longitudinal survey.

Although a few hypotheses have been proposed to explain the association between educational attainment and health, empirical studies have paid little attention to the underlying mechanisms. Education is a reliable predictor of future employment and income (6). Well-educated and wealthy individuals have more financial resources to invest in their health than individuals who are poor, and are thus likely to be healthier (direct income hypothesis) (6, 15). In contrast, some scholars argue that a person’s health status is determined by the distribution of income within a society rather than the absolute level of income (income inequality hypothesis) (16). Having a lower income level or being unemployed causes stress. According to the allostatic load hypothesis, stress is the primary mediator between SEP and health (17). Unlike the abovementioned hypotheses, the time discounting theory postulates that those who are willing to delay gratification invest more resources in their education and health, acquire higher educational attainment, engage in healthier behavior, and thus are well-educated and healthy (18).

Despite these explanatory hypotheses, few previous studies have assessed the effects of potential mediators on the association between educational attainment and midlife health. One study conducted in a Japanese middle-aged population found modest mediating effects of household income level, employment status, and health behaviors with respect to the association between educational inequalities and the incidences of diabetes, stroke, and hypertension (9). In a Danish study of middle-aged adults aged 48–62 years, allostatic load partially mediated the correlation between educational attainment and SRH (19). However, in the case of China, evidence is as yet limited.

We investigated the relationship between educational attainment and the onset of midlife health problems among Chinese population, and evaluated the potential mediating role of other SEP factors, depression, and health behavior as three mechanisms through which educational attainment was associated with midlife health. This study offers two contributions to the field’s existing literature. First, this is among the first longitudinal study to examine the association between educational level and midlife health in the current Chinese population, which is crucial for future older people’s health profile in China. China offers a distinct historical, socioeconomic and demographic context for studying the relationship between educational attainment and midlife health. The current cohort of middle-aged adults (born between 1952 and 1966) witnessed the Great Famine, the cultural revolution, extreme poverty, and political upheaval, which were followed by the country’s opening-up and remarkable economic growth. These life experiences of the current middle-aged cohort differ from those developed in Western ageing societies (8), and they may affect how educational attainment influences health status in midlife. Second, this study provides an initial assessment of the potential mechanisms underlying the association between educational health and midlife health, building on prior hypotheses. We hypothesized that (I) Chinese middle-aged adults with lower educational attainment would experience increased incidences of health problems; and (II) other SEP factors, depression, and health behavior would only minimally mediate the relationship between educational attainment and midlife health among Chinese population.

## 2. Materials and methods

### 2.1. Data and sample

The data for this study were obtained from the China Health and Retirement Longitudinal Study (CHARLS), a nationally representative sample of Chinese adults aged ≥45 years. The baseline survey was conducted in 2011, using multistage probability sampling to select 17,500 individuals in 150 counties across the country. The baseline survey had a response rate of over 80% (94% in rural areas and 69% in urban areas) (20). Follow-up surveys were conducted in 2013, 2015, and 2018.

This study used CHARLS data obtained from the baseline survey to that obtained in 2018 (last wave). There is no consensus on the precise definition of ‘middle age’ or ‘midlife’ in the existing literature (3). This study focused on adults aged 45–59 years at baseline (*N* = 9,534). After excluding those who did not provide information on key variables, a total of 8,050 individuals were included in the analysis. For each health outcome, the following respondents were excluded: those who already reported having health problems in 2011, those who were lost to follow-up in the 2013 survey, and those with missing information on health outcomes or time variables. Finally, the number of respondents analyzed in the study ranged from 4,370 to 6,398, depending on the health outcome.

### 2.2. Measures

#### 2.2.1. Health outcome measures

Health outcome measures included the onset of poor SRH, ADL/IADL limitations, hypertension, dyslipidemia, heart disease, stroke, and multimorbidity, initially diagnosed from 2011 to 2018. For SRH, the survey asked the respondents to choose from among ‘very good’, ‘good’, ‘fair’, ‘poor’, and ‘very poor’ as a response to the question ‘what is the current condition of your health?’ The latter two options indicated poor SRH. A binary variable was constructed for this parameter (1 = SRH poor and 0 = SRH not poor).

The respondents were asked whether they could perform five ADLs (dressing, bathing, eating, transferring, and toileting) and five IADLs (doing housework, cooking, taking medication, shopping, and managing money). For each task, the respondents were given four options: ‘I have no difficulty’, ‘I have difficulty but can still do it’, ‘I have difficulty and need help’, and ‘I cannot do it’. The latter two options indicated a functional limitation in performing the task. The reliability and validity of ADL and IDAL among Chinese population have been supported by previous studies (21, 22). Two binary variables relating to ADL/IADL limitations were created for these parameters (1 = having a functional limitation with reference to performing at least one task and 0 = having no functional limitation to perform any of the tasks).

The CHARLS asked the respondents to report 14 types of NCDs, which included hypertension, dyslipidemia, diabetes, cancer, chronic lung diseases, liver diseases, heart diseases, stroke, kidney diseases, digestive diseases, mental disorders, memory-related diseases, arthritis, and asthma. For each type of NCD, the respondents were asked whether they were diagnosed with the condition by a doctor. It should be noted that the CHARLS did not specify diagnostic criteria for self-reported diseases. This study considered four major NCDs in China, namely hypertension, dyslipidemia, heart disease, and stroke. According to the Global Burden of Disease 2019, stroke and ischemic heart disease were the top two leading causes of death and increased disability-adjusted life years among middle-aged Chinese adults; high systolic blood pressure and high low-density lipoprotein (LDL) cholesterol were the leading metabolic risk factors of the two conditions mentioned above (23). A binary variable was created for each condition (1 = diagnosed with the condition and 0 = was not diagnosed). Multimorbidity was defined as the concurrent occurrence of at least two chronic conditions out of the 14 NCDs in a single patient. A binary variable was constructed accordingly (1 = having multimorbidity and 0 = not having multimorbidity).

#### 2.2.2. Educational level

The survey asked the respondents to choose their final educational attainment from among (I) illiterate, (II) did not finish primary school but capable of reading and/or writing, (III) *Sishu* or home school, (IV) elementary school, (V) junior high school, (VI) high school, (VII) vocational school, (VIII) two-or three-year college or associate degree, (IX) four-year college or bachelor’s degree, (X) masters’ degree, and (XI) doctoral degree. These eleven categories were classified into three educational levels: limited education (I-II), primary school and junior high school (III-V), and high school and above (VI-XI). The ridit score of education attainment was calculated for each educational level. We ordered educational levels from the highest to the lowest, then calculated ridit scores at each level as the proportion of individuals with lower educational level (s) plus one-half of the proportion of individuals in the category itself (24). For example, provided that 20, 30, and 50% of the respondents have high, middle, and low educational levels, the ridit scores for each educational level will be 0.10 (0.2/2; high), 0.35 (0.2 + 0.3/2; middle), and 0.75 (0.2 + 0.3 + 0.5/2; low), respectively (9). A higher ridit score indicates a lower educational level. In regression analysis, the ridit score was used as a continuous variable.

#### 2.2.3. Mediators

This study analyzed a variety of potential mediators such as other SEP indicators, depression, and health behavior at baseline. In addition to educational attainment, this study analyzed household income and occupation as two additional important SEP indicators. We used household spending as a proxy for household income, because household spending may more accurately reflect the standard of living than household income and it can be applied to all the members of a household including women who may not have an income source or who are not the primary earners in the family (6, 9). The self-reported amount was divided by the square root of the number of household members to adjust for household size. After adjusting for household size, a binary variable of ‘low income’ was created by assigning a score of 1 to respondents whose household spending fell into the lowest quartile; a score of 0 was applied to everyone else.

The self-reported job descriptions in CHALRS were coded into five occupation categories using the Erikson and Golthorpe and Portocarero Class Categories: managers and professionals, self-employed, agricultural workers, manual workers, and unemployed (25). This study considered ‘managers and professionals’ as the reference group.

In this study, increase in depressive symptomatology was used as a measure of baseline depression. The CHARLS survey used a short form of the Centre for Epidemiological Studies Depression Scale (CES-D 10) to detect risk of depression in the general population (26). The reliability and validity of CESD-10 among Chinese population have been supported by previous studies (27, 28). It should also be noted that despite good reliability and validity, the depressive symptomatology measured by the CES-D 10 is not equivalent to a diagnosis of depression. When scoring the CES-D 10, a response was given a value of 0, 1, 2, or 3 depending on whether the item was worded positively or negatively. The final score was calculated using the sum of ten items. Because the scoring protocol required that the CES-D data be at least 80% complete, CES-D scores were not calculated for respondents who did not provide responses for two or more items (8). The range of possible scores was 0 to 30, with higher scores indicating the presence of more symptomatology. Respondents with CES-D 10 scores of 12 and higher were considered to have symptoms of depression, because this threshold has reasonable levels of sensitivity and specificity for identifying possible depression cases among Chinese populations (29). Accordingly, a binary variable was constructed for baseline depression (1 = having depression at baseline; 0 = no baseline depression).

This study examined the status of smoking and alcohol drinking as two important health behaviors. CHARLS survey asked respondents if they had ever smoked and, if so, whether they still smoked or had completely quitted. Based on the answers to the two questions, smoking status was classified as ‘current smoker’, ‘former smoker’, and ‘never smoker’. Alcohol drinking status was classified as ‘current heavy drinker’ (consumed ≥5 drinks per day), ‘current light drinker’ (consumed <5 drinks per day), and ‘former or never drinker’, which is consistent with the classification defined by the National Institute on Alcohol Abuse and Alcoholism (30). The reference groups were ‘never smoker’ and ‘former or never drinker’ for smoking and alcohol drinking status, respectively.

#### 2.2.4. Covariates

Following previous studies (8, 12, 31), the covariates considered in this study included age, sex, marital status, urban–rural residence, and the type of social medical insurance schemes at baseline. Sex (1 = female; 0 = male), marital status (1 = married; 0 = never married, widowed, separated, or divorced), and urban–rural residence (1 = urban China; 0 = rural China) were dichotomized. Social medical insurance scheme was categorized into six types (1 = Urban Employee Basic Medical Insurance; 2 = Urban Resident Basic Insurance; 3 = New Rural Cooperative Medical Scheme; 4 = Urban and Rural Resident Medical Insurance; 5 = Government Medical insurance [*Gong Fei*]; 6 = Uninsured). Age was defined as a continuous variable ranging from 45 to 59 years.

#### 2.2.5. Time variables

Time variables were used in the Cox proportional hazard model analysis. To construct the time variables for each health outcome, we calculated the follow-up time as the number of months from the baseline interview date to the follow-up interview date where the first self-report of each health outcome occurred. If censoring occurred when a participant dropped out or did not report a health outcome by the end of the study, the follow-up time was computed as the number of months from the baseline interview date to the date of the respondent’s last interview.

### 2.3. Statistical analysis

For the descriptive analysis, we depicted the key characteristics of the study sample at baseline, and analyzed the trends in the numbers of respondents who reported the onset of each health outcome from 2011 to 2018 at the three different educational levels. The statistical significance of the trends was examined by *p* for trend, based on an extension of the Wilcoxon rank-sum test (32).

Using the Baron and Kenny method, we assessed the mediating effects of other baseline SEP indicators, depression, and health behaviors with five Cox proportional hazards models (33). All mediators and covariates that are derived from baseline data are assumed to be time-constant in our analysis. In Model 1, we first used the ridit score of the educational level only to explain the incidence of each health outcome. We then examined the correlation between educational level, other SEP indictors, depression, and health behavior. Subsequently, we estimated Models 2 to 5 to explain the hazard ratios of each health outcome with respect to the ridit score of educational level and to evaluate the individual and aggregate mediating effects of these mediators. Model 2 added baseline low income and occupation to Model 1; Model 3 added baseline depression to Model 1; Model 4 added baseline smoking and alcohol drinking status to Model 1; and Model 5 added all these mediators to Model 1.

The relative index of inequality (RII) by educational level in health outcome indicates the ratio of the odds or hazards of each outcome at the lowest educational level (with the ridit score = 1) to those at the highest level (with the ridit score = 0). The RII can be derived by computing the exponent of the estimated coefficient of the ridit score of the education level in regression models, as in the cases of the odds ratio (OR) and hazards ratio (HR) (34, 35). This approach has been widely used in previous studies on the association between educational inequalities and health outcomes (9, 36).

The Cox proportional hazard model takes account of the passage of time before the onset of each health outcome, and it works with censored data which is generated when the exact time-to-event for an interested observation is unknown (37, 38). The attenuation rate of the HRs (39) from Models 1 to 5 was used to estimate the extent to which other baseline SEP variables, depression, and health behaviors collectively mediated the educational inequality in midlife health. The Stata software package (Release 16) was used for all the statistical analyses.

## 3. Results

The key characteristics of the study sample at baseline have been summarized in Table 1. Among the total study population, education level was reported as limited education, primary and junior high schools, and high school by 35.6, 47.0, and 17.4% of the respondents, respectively. Table 2 shows the incidence of each health outcome by educational level from 2011 to 2018. Without controlling for any factor, a higher educational level was associated with lower risks of developing poor SRH, multimorbidity, ADL/IADL limitations, and hypertension, whereas the onset of dyslipidemia was positively associated with a higher educational level. The incidences of heart disease and stroke were not associated with educational attainment.

**Table 1:** Key characteristics of the study sample at baseline by educational level.

Variable	Full sample	Limited education		Primary and junior high schools		High school and above	
	*N*	*N*	(%)	*N*	(%)	*N*	(%)
**Low income**							
Yes	1754	820	(28.7)	750	(19.8)	184	(13.1)
No	6,296	2,042	(71.3)	3,035	(80.2)	1,219	(86.9)
**Occupation**							
Managers and professionals	469	13	(0.5)	121	(3.2)	335	(23.9)
Self-employed	823	196	(6.9)	469	(12.4)	158	(11.2)
Agricultural workers	3,531	1,696	(59.3)	1,583	(41.8)	252	(18.0)
Manual workers	1,474	355	(12.3)	810	(21.4)	309	(22.0)
Unemployed	1,753	602	(21.0)	802	(21.2)	349	(24.9)
**Depression**							
Yes	1,922	956	(33.4)	806	(21.3)	160	(11.4)
No	6,128	1,906	(66.6)	2,979	(78.7)	1,243	(88.6)
**Smoking status**							
Current	2,477	626	(21.9)	1,393	(36.8)	458	(32.6)
Former	528	146	(5.1)	266	(7.0)	116	(8.3)
Never	5,045	2090	(73.0)	2,126	(56.2)	829	(59.1)
**Alcohol drinking status**							
Current heavy	1,248	309	(10.8)	690	(18.2)	249	(17.8)
Current light	1,585	410	(14.3)	802	(21.2)	373	(26.6)
Former or never	5,217	2,143	(74.9)	2,293	(60.6)	781	(55.6)
**Social medical insurance scheme**							
Urban Employee Basic Medical Insurance	816	37	(1.3)	299	(7.9)	480	(34.2)
Urban Resident Basic Medical Insurance	360	68	(2.4)	185	(4.9)	107	(7.6)
New Rural Cooperative Medical Scheme	6,004	2,504	(87.5)	2,917	(77.1)	583	(41.6)
Urban and Rural Resident Medical Insurance	100	27	(0.9)	57	(1.5)	16	(1.1)
Government Medical Insurance (*Gong Fei*)	115	4	(0.1)	24	(0.6)	87	(6.2)
Uninsured	655	222	(7.8)	303	(8.0)	130	(9.3)
**Urban–rural residence**							
Rural	4,670	2061	(72.0)	2,149	(56.8)	460	(32.8)
Urban	3,380	801	(28.0)	1,636	(43.2)	943	(67.2)
**Sex**							
Female	4,363	2087	(73.0)	1,690	(44.7)	585	(41.7)
Male	3,687	774	(27.0)	2094	(55.3)	818	(58.3)
**Marital status**							
Yes	7,578	2,637	(92.1)	3,612	(95.4)	1,329	(94.7)
No	472	225	(7.9)	173	(4.6)	74	(5.3)
Age (years)	8,050	2,862		3,785		1,403	
		*M*	(53.6)	*M*	(51.5)	*M*	(51.4)
		*SD*	(4.3)	*SD*	(4.5)	*SD*	(3.9)
**Attrition number**							
2013	1,067	323	(11.3)	493	(13.0)	251	(17.9)
2015	623	167	(6.6)	276	(7.9)	180	(15.6)
2018	544	187	(7.9)	254	(8.4)	103	(10.6)

The order of the social medical insurance schemes simply follows that used presented in the CHARLS survey.

**Table 2:** Incidence (%) of each health outcome over the seven years of follow-up (2011 to 2018).

Health outcome	All	Limited education	Primary and middle schools	High school and above	*p* for trend	*N*
Poor SRH^a^	29.2	37.9	27.3	18.3	<0.001	4,909
Multimorbidity	43.7	45.7	43.3	40.4	0.016	4,370
ADL^b^ limitation	7.3	10.7	5.6	4.3	<0.001	6,226
IADL^c^ limitation	19.5	29.6	16.2	8.5	<0.001	5,996
Hypertension	22.4	23.3	22.9	18.9	0.028	5,088
Dyslipidemia	18.6	16.4	18.4	24.4	<0.001	5,574
Heart diseases	12.9	12.1	13.0	14.6	0.070	5,822
Stroke	6.0	6.1	5.6	6.1	0.918	6,398

a Self-rated health.

b Activities of daily living.

c Instrumental activities of daily living.

**Figure 1:**
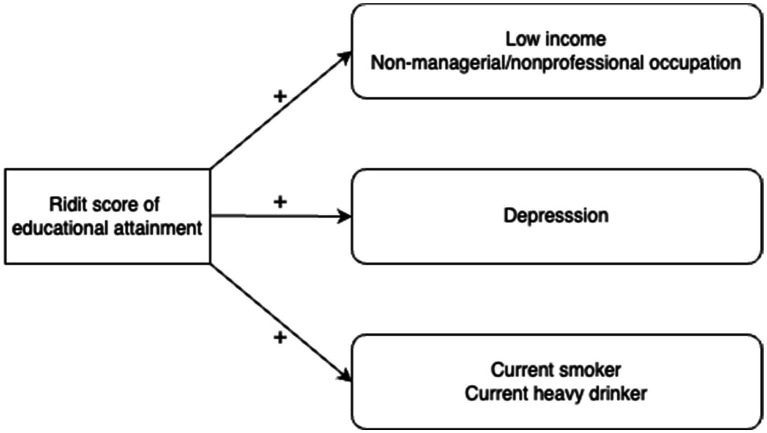
Directed acyclic graph for the relationship between the ridit score of educational attainment and each mediator.

We then confirmed significant correlations between the ridit score of educational attainment and each potential mediator (see Table S1 in the Supplementary material). The ridit score of educational attainment had positive and close correlations with low income, non-managerial or nonprofessional occupation, depression, current smoker, and current heavy drinker at baseline, suggesting the roles of these factors as mediators of the impact of educational attainment on health outcomes (see Figure 1). The magnitudes of their correlations were relatively modest, ranging from 0.083 to 0.185. Therefore, we do not need to be seriously concerned about any unmeasured factor that would closely link educational attainment and each potential mediator.

The results of the Cox proportional hazard model analyses are presented in Table 3. From the results in Model 1, which controlled for baseline covariates but did not include any potential mediators, we observed significant associations at the 0.1% significance level for various health indicators. Specifically, the RII for poor SRH was 2.17 (95% confidence interval [CI]: 1.74, 2.70), for ADL limitation was 2.15 (95% CI: 1.42, 3.26), and for IADL limitation was 3.84 (95% CI: 2.98, 4.94); whereas, the RII for dyslipidemia was 0.52 (95% CI: 0.40, 0.68), and for heart disease was 0.55 (95% CI: 0.40, 0.74). Thus, higher educational attainment was associated with lower risks of poor SRH and ADL/IADL limitations, while it was associated with increased risks of developing dyslipidemia and heart disease. Multimorbidity, hypertension, and stroke were not associated with educational attainment.

**Table 3:** Estimated hazard ratios of each health outcome, respectively, by educational level: Cox proportional hazard models^a^.

	Model 1^b^		Model 2^c^		Model 3^c^		Model 4^c^		Model 5^c^	
	HR^d^	95% CI^e^	HR	95% CI	HR	95% CI	HR	95% CI	HR	95% CI
Poor SRH^f^	2.17^***^	(1.74, 2.70)	2.06^***^	(1.65, 2.59)	2.01^***^	(1.61, 2.51)	2.16^***^	(1.74, 2.70)	1.94^***^	(1.55, 2.44)
Multimorbidity	1.13	(0.94, 1.37)	1.12	(0.92, 1.36)	1.07	(0.88, 1.30)	1.13	(0.93, 1.37)	1.07	(0.88, 1.30)
ADL^g^ limitation	2.15^***^	(1.42, 3.26)	2.12^***^	(1.40, 3.22)	1.84^**^	(1.21, 2.80)	2.12^***^	(1.40, 3.23)	1.84^**^	(1.21, 2.80)
IADL^h^ limitation	3.84^***^	(2.98, 4.94)	3.42^***^	(2.66, 4.40)	3.49^***^	(2.70, 4.50)	3.85^***^	(2.99, 4.97)	3.16^***^	(2.44, 4.08)
Hypertension	1.15	(0.90, 1.47)	1.15	(0.90, 1.48)	1.12	(0.87, 1.43)	1.13	(0.89, 1.45)	1.11	(0.86, 1.43)
Dyslipidemia	0.52^***^	(0.40, 0.68)	0.55^***^	(0.42, 0.73)	0.49^***^	(0.38, 0.65)	0.52^***^	(0.40, 0.68)	0.53^***^	(0.40, 0.70)
Heart disease	0.55^***^	(0.40, 0.74)	0.54^***^	(0.39, 0.74)	0.51^***^	(0.38, 0.70)	0.54^***^	(0.40, 0.74)	0.51^***^	(0.37, 0.69)
Stroke	0.80	(0.52, 1.24)	0.82	(0.53, 1.26)	0.73	(0.47, 1.13)	0.80	(0.52, 1.23)	0.75	(0.48, 1.16)

a Full estimation results are presented in Table S2 in the Supplementary file.

b Adjusted for age, sex, marital status, urban–rural residence, and type of social medical insurance schemes at baseline.

c Further adjusted for low income and occupation (Model 2), depression (Model 3), smoking and alcohol drinking status (Model 4), and all the factors (Model 5).

d Hazard ratio, which indicates the relative index of inequality (RII) of educational level.

e Confidence interval.

f Self-rated health.

g Activities of daily living.

h Instrumental activities of daily living.

^***^p < 0.001, ^**^p < 0.01, ^*^p < 0.05.

When adding all five mediators in Model 5, the HRs for the ridit score of educational level remained well above one for poor SRH, ADL, and IADL limitations (*p* < 0.001 for all), while it remained well below one for dyslipidemia and heart disease (*p* < 0.001 for both). Furthermore, the estimated HRs changed minimally or moderately from Model 1 to 5, indicating the limited role played by the potential mediators in effect of educational inequalities on midlife health.

We then quantified the magnitude of the mediated effect by measuring the attenuation rate of HR from Model 1 to Model 5; the attenuation rate was calculated as (HR in Model 1 - HR in Model 5)/(HR in Model 1–1) × 100% (9, 40), and 95% CIs were computed by bootstrapping estimations (with 3,000 replications). Table 4 shows that the rate of attenuation varied from 2.1% (dyslipidemia) to 27.0% (ADL limitation), while adding the potential mediators amplified educational inequality by 8.9% for heart disease.

**Table 4:** Estimated hazard ratios of each health outcome with respect to the ridit score of the educational level.

	Model 1^a^		Model 5^b^		Attenuation in HR^c^		*N*
	HR^d^	95% CI^e^	HR	95% CI	(%)	95% CI	
Poor SRH^f^	2.17^***^	(1.74, 2.70)	1.94^***^	(1.55, 2.44)	19.7	(18.8, 19.9)	4,909
Multimorbidity	1.13	(0.94, 1.37)	1.07	(0.88, 1.30)			4,370
ADL^g^ limitation	2.15^***^	(1.42, 3.26)	1.84^**^	(1.21, 2.80)	27.0	(26.0, 28.0)	6,226
IADL^h^ limitation	3.84^***^	(2.98, 4.94)	3.16^***^	(2.44, 4.08)	23.9	(23.7, 24.5)	5,996
Hypertension	1.15	(0.90, 1.47)	1.11	(0.86, 1.43)			5,088
Dyslipidemia	0.52^***^	(0.40, 0.68)	0.53^***^	(0.40, 0.70)	2.1	(1.6, 2.5)	5,574
Heart diseases	0.55^***^	(0.40, 0.74)	0.51^***^	(0.37, 0.69)	−8.9	(−9.6, −8.4)	5,822
Stroke	0.80	(0.52, 1.24)	0.75	(0.48, 1.16)			6,398

a Adjusted for age, sex, marital status, urban–rural residence, and type of social medical insurance schemes at baseline.

b Adjusted for other SEP variables, depression, and health behavior, as well as age, sex, marital status, urban–rural residence, and type of social medical insurance schemes at baseline.

c Attenuation rate = (HR in Model 1 - HR in Model 5)/(HR in Model 1–1) × 100%; 95% CIs were computed by bootstrapping estimations (with 3,000 replications).

d Hazard ratio, which indicates the relative index of inequality (RII) of educational level.

e Confidence interval.

f Self-rated health.

g Activities of daily living.

h Instrumental activities of daily living.

^***^p < 0.001, ^**^p < 0.01.

Table S2 (in the Supplementary material) summarizes the results of the Cox proportional hazard model analyses. Results from Model 5 showed varying effects across mediators. Low income was associated with a higher incidence of IADL limitation, but not with any other health outcome. Compared to managerial and professional occupations, agricultural work and unemployment were associated with higher incidences of ADL/IADL limitations. Unemployment was also associated with a heightened risk of poor SRH. However, in the case of dyslipidemia, only manual work was associated with a lower risk of developing the condition. Baseline depression was associated with increased incidences of all the adverse health outcomes except for hypertension. Neither smoking nor alcohol drinking status were found to be strong predictors of the outcomes. Only in the case of poor SRH, current light drinkers showed a decreased risk compared to never drinkers. We further assessed potential sex and urban–rural disparities, but found insignificant results.

## 4. Discussion

We examined the effect of educational inequalities on health among the Chinese middle-aged population using data from a four-wave longitudinal nationwide survey. Unlike previous studies, this study focused on midlife (a pivotal stage in a person’s life), employed Cox proportional hazard models to analyze data over a 7-year follow-up period, and assessed a variety of potential mediators that may underlie the association between education and health. The following are some of the key findings and their implications.

First, our study uncovered that educational inequalities were significantly associated with poor SRH, and ADL/IADL limitations. Even after adjusting for other baseline SEP indicators, depression, and health behavior, lower educational attainment correlated significantly with the onset of poor SRH and ADL/IADL limitations. According to the efficient producer hypothesis, better-educated individuals are more efficient at making health investments than less educated individuals (40). For instance, a person’s cognitive functioning is affected by the knowledge and skills acquired in school, which makes them more responsive to health education information, better at communicating with health service providers, and navigating complex treatment regimens (6). Furthermore, no sex or urban–rural residence differences were discovered in this study. Our findings reveal the importance of removing educational disparities in childhood and early adulthood for mitigating health inequalities in middle and late life, regardless of sex or urban–rural residence.

Second, higher educational attainment was associated with heightened incidences of dyslipidemia and heart disease, but not with that of hypertension or stroke. While being a protective factor for reducing the odds of poor SRH and ADL/IADL limitations, educational attainment was a risk factor for the onset of dyslipidemia and heart disease. This seemingly counterintuitive phenomenon violates the predictions as per the efficient producer hypothesis. However, our study found that higher educational attainment raised the risks of smoking and heavy drinking, suggesting that education may have at least partly adverse impact on midlife health in China.

In this regard, previous studies in China, Japan, and South Korea have indicated that higher educational attainment is a risk factor for dyslipidemia (9, 41, 42). This could be because those with higher educational levels are more likely to be sedentary, consume high-fat food, and have high levels of workplace stress (9, 41, 42). In our study, manual workers, similar to those with low educational attainment, also had a lower incidence of dyslipidemia than professionals and managers. The observed SEP disparities in the onset of dyslipidemia may be explained by the nature of work, lifestyle, and diet. It is also surprising to see a positive association between higher educational attainment and increased risk of heart disease. Previous research has shown mixed results: results from a study in Tibetan Chinese adults (43) support our finding, whereas those from a study in black Americans (44) contradict findings from our study. The disparity in economic development between the populations in previous studies and ours could explain this inconsistency.

Over the past four decades, China has experienced rapid economic growth which has profound public health implications, particularly for the middle class. Technology innovation and economic development have altered the relative price of consuming and expending calories (45). Technological advancement has lowered food prices and the time costs of food preparation (45). In the meantime, economic development has changed the nature of work: workers were previously paid to exert their bodies due to the strenuous nature of their work; however, with sedentary jobs, workers now have to pay for leisure-based exercise as a substitute for on-the-job exercise (45). Other emerging economies such as India have also reported that the middle class is vulnerable to obesity and cardiovascular disease (46). Middle-aged adults with higher educational attainment have traditionally been defined as a privileged group. However, our findings show that this group is at risk for dyslipidemia and heart disease. Thus, public health authorities should take timely action to promote healthy diets and physical activities among middle-aged adults with higher educational attainment. With the advancement of economic growth, a deterioration in cardiometabolic health could be foreseen even among lower SEP groups, and the middle class may merely be a precursor to this trend. Accordingly, public health authorities need to act quickly to prevent this from happening.

Third, our study indicates minimal to moderate effects of other SEP indicators, depression, and health behaviors on educational inequalities in midlife health. The limited effects of income level and occupation cast doubt on the hypothesized mechanisms linking education to health; these mechanisms focus on material resource components. The minimal mediating effect of smoking and alcohol drinking status also failed to corroborate the time discounting theory, which predicts that those who invest more resources in their education will also avoid unhealthy behaviors.

An increase in depression symptoms at baseline was a significant predictor of all health outcomes in this study. This parameter showed greater, albeit modest, mediating effects compared to other SEP factors and health behaviors. In response to chronic stress and challenging life events, different physiological systems interact at various degrees of activity, and when external stressors exceed one’s ability to cope with the stress, allostatic overload occurs (45). According to the allostatic load hypothesis, an individual under prolonged or recurring stress exhibits a higher rate of depreciation of health capital than someone who lives a less stressful life, even at the same biological age (17). As a result, even if the distressed person invests the same amount of resources for health maintenance as a relaxed person, their investment in health is less beneficial, and their level of health is lower (17). Public health authorities should pay attention to stressors in middle age and earlier life. Given the minimal to moderate roles of other SEP factors, depression, and health behavior on midlife health, our study stresses the importance of achieving educational equality and suggests a spillover benefit of educational policy on population health.

Fourth, an interesting finding emerges from our analysis, revealing an amplified educational inequality in relation to heart diseases after adding potential mediators into the model. As shown in Table S2 in the Supplementary material, depression appears as a key mediator that affected the change in the strength of association between the ridit score of educational attainment and the onset of having heart diseases. This observation implies that depression may act as a ‘suppressor’, whereby the inclusion of the suppressor leads to an increase in the size of the main effect (47). This occurs because the mediated indirect effect and the direct effect have opposite signs (47). Following Hair et al. diagnostic procedure (48), we identified significant and positive correlations between the ridit score of educational attainment and depression (see Table S1 in the Supplementary material), as well as between depression and the onset of heart diseases (see Table S2 in the Supplementary material). However, we observed a significant and negative correlation between the ridit score of educational attainment and the onset of heart diseases (see Table 3). These findings suggest the presence of a competitive mediation model in this scenario (48), where the indirect effect through depression and the direct effect from educational inequality to the onset of heart diseases are both significant but point in opposite directions. Future studies could investigate the positive relationship between depression and the onset of heart diseases in midlife.

This study has five limitations which should be given due attention. First, this study employed the CES-D 10 to measure baseline depression, rather than utilizing an objective measurement of ‘allostatic load’. Future studies should employ biomarkers and clinical criteria which are widely used in epidemiological studies (49). Second, we only considered the onset of health problems, and did not include data on disease progression. A previous study suggests that the predictive ability of education attainment may be different with reference to the prediction of the onset and progression of functional limitations and chronic diseases (50). Third, due to data availability issues, our study only assessed selected mediators, leaving others unexamined. Cognitive functioning, living conditions, physical activity, and dietary habits are examples of additional mediators that can be included (6, 9). In addition, we applied the Baron and Kenney’s analysis strategy for mediation analysis which cannot quantify mediating effects, although we computed the attenuation rate of HRs as a proxy for the magnitude of mediating effects. Future study may use other techniques, such as structural equation modelling, to directly quantify the indirect effect of education on midlife health through each mediator. Fourth, we only examined the correlation between education attainment and midlife health. We cannot rule out the case that illness in childhood and adolescence could limit educational attainment and predispose a person to diseases in adulthood, leading to a health selection effect on health inequalities (6). More research is needed to further examine the causation from educational attainment to midlife health. Fifth, the time constant assumption of all mediators may have overlooked their dynamic interactions with education and health, which could lead to biased estimation, although our assumption test and robustness assessment (available upon request) suggested that such a risk was small.

In conclusion, our findings suggest that educational attainment is an important predictor of the incidence of the key midlife health problems, with significant mediating effects from other SEP factors, depression, and health behavior. Furthermore, while a higher level of education was revealed to be a protective factor against the onset of poor SRH and ADL/IADL limitations, it was found to be a risk factor for dyslipidemia and heart disease. Political commitment to eliminating educational disparities in childhood and early adulthood also holds potential to improve midlife health. Public health practitioners in China should improve their health promotion strategies to target middle-aged adults with higher educational attainment.

## Data availability statement

Publicly available datasets were analyzed in this study. This data can be found at: http://charls.pku.edu.cn/en.

## Ethics statement

Ethical review and approval was not required for the study on human participants in accordance with the local legislation and institutional requirements. Written informed consent for participation was not required for this study in accordance with the national legislation and the institutional requirements.

## Author contributions

RP organized this research project and conceptualized and designed the study, collected the data and performed a formal analysis, and prepared the initial manuscript. TO reviewed and edited the manuscript and acquired the required funds. All authors contributed to the article and approved the submitted version.

## Funding

This research was funded by Japan Society for the Promotion of Science (JSPS; grant number 20K01722). The JSPS played no role in the design of the study, collection, analysis, interpretation of data, or in writing the manuscript.

## Conflict of interest

The authors declare that the research was conducted in the absence of any commercial or financial relationships that could be constructed as a potential conflict of interest.

## Publisher’s note

All claims expressed in this article are solely those of the authors and do not necessarily represent those of their affiliated organizations, or those of the publisher, the editors and the reviewers. Any product that may be evaluated in this article, or claim that may be made by its manufacturer, is not guaranteed or endorsed by the publisher.
