# Supplementary material for: Education level as a predictor of the onset of health problems among China’s middle-aged population: Cox regression analysis
Source: Front Public Health. 2023 Jul 14;11:1187336. doi: 10.3389/fpubh.2023.1187336 (PMC10379631; doi:10.3389/fpubh.2023.1187336)
Supplement: Supplementary file 1 [file Table_1.docx]

Supplementary Material

Education level as a predictor of the onset of health problems among China’s middle-aged population: Cox regression analysis

Ruru Ping, Takashi Oshio^*^

*** Correspondence:** Takashi Oshio; [oshio@ier.hit-u.ac.jp](mailto:oshio@ier.hit-u.ac.jp)

**Table S1.** Pairwise Spearman correlation between the ridit score of educational attainment and mediators

|  | 1 | 2 | 3 | 4 | 5 | 6 |
| --- | --- | --- | --- | --- | --- | --- |
| 1. Ridit score of educational attainment | 1 |  |  |  |  |  |
| 2. Low income | 0.136^***^ | 1 |  |  |  |  |
| 3. Non-managerial, nonprofessional occupation | 0.083^***^ | -0.018 | 1 |  |  |  |
| 4. Depression | 0.185^***^ | 0.057^***^ | 0.067^***^ | 1 |  |  |
| 5. Current smoker | 0.133^***^ | 0.023^**^ | 0.111^***^ | 0.058^***^ | 1 |  |
| 6. Current heavy drinker | 0.143^***^ | 0.046^***^ | 0.140^***^ | 0.083^***^ | 0.439^***^ | 1 |

^**^*p* < .01, ^***^*p* < .001

**Table S2.** Estimated hazard ratios of each health outcome by educational level and baseline SES, depression, and health behaviors^a^

|  | Model 1 | Model 2 | Model 3 | Model 4 | Model 5 |
| --- | --- | --- | --- | --- | --- |
|  | HR^b^ (95%CI) | HR (95%CI) | HR (95%CI) | HR (95%CI) | HR (95%CI) |
| Poor self-rated health |  |  |  |  |  |
| Ridit score of three educational levels | 2.17^***^ (1.74, 2.70) | 2.06^***^ (1.65, 2.59) | 2.01^***^ (1.61, 2.51) | 2.16^***^ (1.74, 2.70) | 1.94^***^ (1.55, 2.44) |
| Low income |  | 1.05 (0.93, 1.18) |  |  | 1.05(0.93, 1.18) |
| Occupation^c^ |  |  |  |  |  |
| Self-employed |  | 1.03 (0.74, 1.44) |  |  | 1.02 (0.73, 1.43) |
| Agricultural workers |  | 1.51^**^ (1.12, 2.04) |  |  | 1.43^*^ (1.06, 1.94) |
| Manual workers |  | 1.03 (0.75, 1.40) |  |  | 1.01 (0.74, 1.39) |
| Unemployed |  | 1.77^***^ (1.30, 2.40) |  |  | 1.66^***^ (1.22, 2.27) |
| Depression |  |  | 1.84^***^ (1.64, 2.06) |  | 1.79^***^ (1.59, 2.01) |
| Smoking status^d^ |  |  |  |  |  |
| Current smoker |  |  |  | 1.03 (0.88, 1.21) | 1.02 (0.87, 1.20) |
| Former smoker |  |  |  | 1.15 (0.91, 1.45) | 1.16 (0.92, 1.46) |
| Alcohol drinking status^e^ |  |  |  |  |  |
| Current heavy drinker |  |  |  | 0.87 (0.74, 1.03) | 0.91 (0.77, 1.07) |
| Current light drinker |  |  |  | 0.82^**^ (0.71, 0.94) | 0.82^**^ (0.71, 0.95) |
| Multimorbidity |  |  |  |  |  |
| Ridit score of three educational levels | 1.13 (0.94, 1.37) | 1.12 (0.92, 1.36) | 1.07 (0.88, 1.30) | 1.13 (0.93, 1.37) | 1.07 (0.88, 1.30) |
| Low income |  | 0.98 (0.89, 1.09) |  |  | 0.97 (0.87, 1.07) |
| Occupation |  |  |  |  |  |
| Self-employed |  | 0.91 (0.72, 1.15) |  |  | 0.89 (0.70, 1.12) |
| Agricultural workers |  | 1.05 (0.84, 1.30) |  |  | 1.00 (0.80, 1.24) |
| Manual workers |  | 0.87 (0.70, 1.08) |  |  | 0.86 (0.69, 1.07) |
| Unemployed |  | 1.13 (0.90, 1.40) |  |  | 1.05 (0.84, 1.32) |
| Depression |  |  | 1.48^***^ (1.33, 1.64) |  | 1.46^***^ (1.31, 1.62) |
| Smoking status |  |  |  |  |  |
| Current smoker |  |  |  | 1.04 (0.91, 1.19) | 1.03 (0.90, 1.18) |
| Former smoker |  |  |  | 1.28^*^ (1.05, 1.55) | 1.27^*^ (1.04, 1.54) |
| Alcohol drinking status |  |  |  |  |  |
| Current heavy drinker |  |  |  | 0.93 (0.81, 1.07) | 0.96 (0.84, 1.11) |
| Current light drinker |  |  |  | 0.86^*^ (0.76, 0.98) | 0.87^*^ (0.77, 0.99) |
| ADL problems |  |  |  |  |  |
| Ridit score of three educational levels | 2.15^***^ (1.42, 3.26) | 2.12^***^ (1.40, 3.22) | 1.84^**^ (1.21, 2.80) | 2.12^***^ (1.40, 3.23) | 1.84^**^ (1.21, 2.80) |
| Low income |  | 0.91 (0.73, 1.12) |  |  | 0.89 (0.72, 1.10) |
| Occupation |  |  |  |  |  |
| Self-employed |  | 2.75 (0.82, 9.17) |  |  | 2.62 (0.78, 8.75) |
| Agricultural workers |  | 4.37^*^ (1.38, 13.79) |  |  | 3.93^*^ (1.24, 12.44) |
| Manual workers |  | 2.74 (0.84, 8.90) |  |  | 2.62 (0.81, 8.51) |
| Unemployed |  | 8.94^***^ (2.81, 28.41) |  |  | 7.82^***^ (2.45, 24.94) |
| Depression |  |  | 2.19^***^ (1.81, 2.65) |  | 2.07^***^ (1.71, 2.50) |
| Smoking status |  |  |  |  |  |
| Current smoker |  |  |  | 1.06 (0.76, 1.49) | 1.02 (0.74, 1.42) |
| Former smoker |  |  |  | 1.05 (0.65, 1.69) | 1.04 (0.65, 1.67) |
| Alcohol drinking status |  |  |  |  |  |
| Current heavy drinker |  |  |  | 0.85 (0.61, 1.17) | 0.99 (0.72, 1.36) |
| Current light drinker |  |  |  | 0.77 (0.58, 1.02) | 0.84 (0.64, 1.11) |
| IADL problems |  |  |  |  |  |
| Ridit score of three educational levels | 3.84^***^ (2.98, 4.94) | 3.42^***^ (2.66, 4.40) | 3.49^***^ (2.70, 4.50) | 3.85^***^ (2.99, 4.97) | 3.16^***^ (2.44, 4.08) |
| Low income |  | 1.34^***^ (1.19, 1.52) |  |  | 1.33^***^ (1.17, 1.50) |
| Occupation |  |  |  |  |  |
| Self-employed |  | 1.54 (0.91, 2.61) |  |  | 1.50 (0.88, 2.56) |
| Agricultural workers |  | 2.50^***^ (1.52, 4.11) |  |  | 2.33^***^ (1.41, 3.84) |
| Manual workers |  | 1.48 (0.89, 2.47) |  |  | 1.44 (0.87, 2.41) |
| Unemployed |  | 3.04^***^ (1.84, 5.00) |  |  | 2.77^***^ (1.67, 4.58) |
| Depression |  |  | 1.92^***^ (1.70, 2.16) |  | 1.83^***^ (1.62, 2.06) |
| Smoking status |  |  |  |  |  |
| Current smoker |  |  |  | 0.95 (0.79, 1.15) | 0.93 (0.77, 1.13) |
| Former smoker |  |  |  | 0.99 (0.75, 1.31) | 1.02 (0.77, 1.35) |
| Alcohol drinking status |  |  |  |  |  |
| Current heavy drinker |  |  |  | 0.88 (0.73, 1.08) | 0.98 (0.81, 1.19) |
| Current light drinker |  |  |  | 0.89 (0.75, 1.05) | 0.91 (0.77, 1.08) |
| Hypertension |  |  |  |  |  |
| Ridit score of three educational levels | 1.15 (0.90, 1.47) | 1.15 (0.90, 1.48) | 1.12 (0.87, 1.43) | 1.13 (0.89, 1.45) | 1.11 (0.86, 1.43) |
| Low income |  | 1.08 (0.94, 1.23) |  |  | 1.08 (0.94, 1.23) |
| Occupation |  |  |  |  |  |
| Self-employed |  | 0.85 (0.62, 1.18) |  |  | 0.84 (0.61, 1.16) |
| Agricultural workers |  | 0.94 (0.70, 1.26) |  |  | 0.93 (0.69, 1.25) |
| Manual workers |  | 0.81 (0.60, 1.09) |  |  | 0.81 (0.60, 1.09) |
| Unemployed |  | 1.04 (0.76, 1.40) |  |  | 1.02 (0.75, 1.39) |
| Depression |  |  | 1.15 (1.01, 1.31) |  | 1.14 (1.00, 1.30) |
| Smoking status |  |  |  |  |  |
| Current smoker |  |  |  | 1.00 (0.84, 1.19) | 1.00 (0.84, 1.19) |
| Former smoker |  |  |  | 1.15 (0.90, 1.48) | 1.16 (0.90, 1.49) |
| Alcohol drinking status |  |  |  |  |  |
| Current heavy drinker |  |  |  | 1.06 (0.90, 1.26) | 1.09 (0.92, 1.29) |
| Current light drinker |  |  |  | 0.85 (0.73, 1.00) | 0.86 (0.74, 1.01) |
| Dyslipidemia |  |  |  |  |  |
| Ridit score of three educational levels | 0.52^***^ (0.40, 0.68) | 0.55^***^ (0.42, 0.73) | 0.49^***^ (0.38, 0.65) | 0.52^***^ (0.40, 0.68) | 0.53^***^ (0.40, 0.70) |
| Low income |  | 1.02 (0.88, 1.18) |  |  | 1.01 (0.88, 1.17) |
| Occupation |  |  |  |  |  |
| Self-employed |  | 0.75 (0.55, 1.02) |  |  | 0.75 (0.55, 1.02) |
| Agricultural workers |  | 0.76 (0.58, 1.00) |  |  | 0.74^*^ (0.56, 0.98) |
| Manual workers |  | 0.70 (0.53, 0.93) |  |  | 0.70^*^ (0.52, 0.92) |
| Unemployed |  | 0.88 (0.66, 1.16) |  |  | 0.84 (0.63, 1.12) |
| Depression |  |  | 1.27^***^ (1.11, 1.46) |  | 1.27^***^ (1.10, 1.46) |
| Smoking status |  |  |  |  |  |
| Current smoker |  |  |  | 0.98 (0.81, 1.18) | 0.97 (0.80, 1.18) |
| Former smoker |  |  |  | 1.27 (0.98, 1.66) | 1.27 (0.98, 1.66) |
| Alcohol drinking status |  |  |  |  |  |
| Current heavy drinker |  |  |  | 0.88 (0.72, 1.08) | 0.90 (0.73, 1.10) |
| Current light drinker |  |  |  | 0.95 (0.80, 1.12) | 0.96 (0.81, 1.14) |
| Heart diseases |  |  |  |  |  |
| Ridit score of three educational levels | 0.55^***^ (0.40, 0.74) | 0.54^***^ (0.39, 0.74) | 0.51^***^ (0.38, 0.70) | 0.54^***^ (0.40, 0.74) | 0.51^***^ (0.37, 0.69) |
| Low income |  | 1.03 (0.87, 1.22) |  |  | 1.03 (0.87, 1.21) |
| Occupation |  |  |  |  |  |
| Self-employed |  | 1.12 (0.76, 1.66) |  |  | 1.12 (0.75, 1.66) |
| Agricultural workers |  | 1.12 (0.80, 1.61) |  |  | 1.09 (0.76, 1.57) |
| Manual workers |  | 0.83 (0.56, 1.21) |  |  | 0.82 (0.56, 1.20) |
| Unemployed |  | 1.26 (0.87, 1.82) |  |  | 1.22 (0.84, 1.77) |
| Depression |  |  | 1.37^***^ (1.17, 1.61) |  | 1.35^***^ (1.15, 1.59) |
| Smoking status |  |  |  |  |  |
| Current smoker |  |  |  | 1.12 (0.88, 1.42) | 1.11 (0.88, 1.41) |
| Former smoker |  |  |  | 1.36 (0.99, 1.88) | 1.35 (0.97, 1.86) |
| Alcohol drinking status |  |  |  |  |  |
| Current heavy drinker |  |  |  | 1.07 (0.84, 1.35) | 1.11 (0.88, 1.40) |
| Current light drinker |  |  |  | 0.93 (0.76, 1.13) | 0.95 (0.78, 1.16) |
| Stroke |  |  |  |  |  |
| Ridit score of three educational levels | 0.80 (0.52, 1.24) | 0.82 (0.53, 1.26) | 0.73 (0.47, 1.13) | 0.80 (0.52, 1.23) | 0.75 (0.48, 1.16) |
| Low income |  | 1.20 (0.95, 1.51) |  |  | 1.19 (0.95, 1.50) |
| Occupation |  |  |  |  |  |
| Self-employed |  | 0.81 (0.46, 1.45) |  |  | 0.81 (0.45, 1.44) |
| Agricultural workers |  | 1.02 (0.61, 1.69) |  |  | 0.99 (0.59, 1.64) |
| Manual workers |  | 0.77 (0.45, 1.31) |  |  | 0.76 (0.44, 1.30) |
| Unemployed |  | 1.44 (0.87, 2.38) |  |  | 1.37 (0.83, 2.28) |
| Depression |  |  | 1.59^***^ (1.28, 1.98) |  | 1.54^***^ (1.24, 1.90) |
| Smoking status |  |  |  |  |  |
| Current smoker |  |  |  | 1.03 (0.75, 1.42) | 1.02 (0.74, 1.40) |
| Former smoker |  |  |  | 1.23 (0.80, 1.89) | 1.23 (0.80, 1.88) |
| Alcohol drinking status |  |  |  |  |  |
| Current heavy drinker |  |  |  | 1.04 (0.76, 1.43) | 1.13 (0.82, 1.56) |
| Current light drinker |  |  |  | 0.95 (0.72, 1.26) | 1.00 (0.76, 1.32) |

^a^ Adjusted for age, gender, marital status, urban-rural residence, and type of social medical insurance schemes at baseline

^b^ Hazard ratio, which indicates the relative index of inequality (RII) of educational level.

^c^ The reference group was ‘managers and professionals’.

^d^ The reference group was ‘never smoker’.

^e^ The reference group was ‘never drinker’.

^***^*p* < .001, ^**^*p* < .01, ^*^*p* < .05
